# Astrovirus Pathogenesis

**DOI:** 10.3390/v9010022

**Published:** 2017-01-22

**Authors:** Cydney Johnson, Virginia Hargest, Valerie Cortez, Victoria A. Meliopoulos, Stacey Schultz-Cherry

**Affiliations:** 1 Department of Infectious Diseases, St. Jude Children’s Research Hospital, Memphis, TN 38105, USA; Cydney.Johnson@stjude.org (C.J.); Virginia.Hargest@stjude.org (V.H.); Valerie.Cortez@stjude.org (V.C.); Victoria.Meliopoulos@stjude.org (V.A.M.); 2 Department of Microbiology, Immunology, and Biochemistry, University of Tennessee Health Science Center, Memphis, TN 38163, USA

**Keywords:** astrovirus, pathogenesis, animal models, cell culture

## Abstract

Astroviruses are a major cause of diarrhea in the young, elderly, and the immunocompromised. Since the discovery of human astrovirus type 1 (HAstV-1) in 1975, the family *Astroviridae* has expanded to include two more human clades and numerous mammalian and avian-specific genotypes. Despite this, there is still little known about pathogenesis. The following review highlights the current knowledge of astrovirus pathogenesis, and outlines the critical steps needed to further astrovirus research, including the development of animal models of cell culture systems.

## 1. Introduction

**Figure 1:**
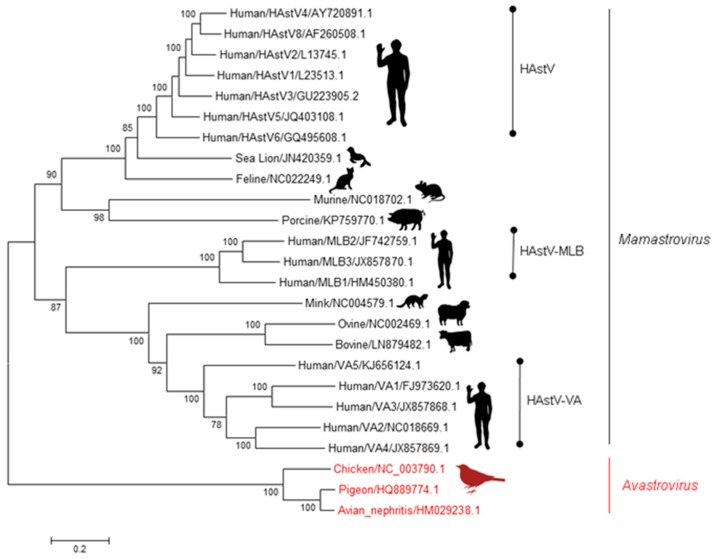
A brief phylogenetic tree of the *Astroviridae* family. Phylogenetic tree with representative *Avastrovirus* (**red**) and *Mamastrovirus* (**black**) genotypes. Using full genome sequences, the evolutionary history was inferred using the Maximum Likelihood method based on the Kimura 2-parameter model. Trees were constructed using 500 bootstrapped replicates, with values above 70 shown. Branch lengths represent the number of substitutions per site. HAstV: Human astrovirus; MLB: Melbourne; VA: Virginia.

Infectious diarrhea is a leading cause of childhood morbidity and mortality worldwide. Human astroviruses (HAstV)—small, non-enveloped positive-sense single-stranded RNA viruses in the *Astroviridae* family—are a leading cause of diarrhea in children, the elderly, and immunocompromised people. They are extremely prevalent, and around 90% of the population 9 years and older has antibodies against HAstV type 1 (HAstV-1) [1,2,3]. Since the initial discovery of HAstV-1 in humans in 1975 [4,5], additional astroviruses have been described and reported in humans [6] as well as in domesticated and wild animals [7,8,9,10,11,12,13,14,15,16,17,18,19,20,21,22,23,24,25]. This broad host range has become increasingly evident, with the viruses divided into two main genera—*Avastrovirus* and *Mamastrovirus*—based on their ability to infect avian and mammalian species, respectively (Figure 1).

Astrovirus infections were thought to be species-specific, yet turkey, chicken, and duck astroviruses share genetic features, indicating that cross-species transmission may be frequent [26]. Poultry abattoir workers were three times more likely to test positive for antibodies against turkey astrovirus than people with no contact with poultry [27], and perhaps the most compelling evidence was the detection of astrovirus strains previously shown to be limited to human infections in non-human primates (NHP) by real-time polymerase chain reaction (RT-PCR) [28]. These studies and others suggest that astroviruses can cross species barriers; however, whether these transmissions result in disease remains unclear.

Despite the high prevalence of astrovirus [29] and the advances made in the identification of novel genotypes, there is still little known about HAstV pathogenesis—especially among the different HAstV genotypes. Previous studies demonstrated that HAstVs increase epithelial cell permeability by disrupting cellular tight junctional complexes [30]. Since the intestinal tract depends on tight junctions to separate the lumen from the basal lamina, the loss of integrity increases ion, solute, and water trafficking across the compartments, reducing the ability of the intestine to reabsorb water and nutrients, leading to diarrhea. Additionally, extra-gastrointestinal astrovirus-associated disease has been reported in animals and humans [31,32,33], which could result from the increased intestinal permeability. The definite mechanisms underlying diarrhea and systemic spread remain an area of continual research; increasing our understanding of astrovirus disease will be integral for future treatment and prevention strategies. Recently, studies in both humans and animals have helped to elucidate the molecular and cellular attributes of astrovirus disease. The following review highlights these findings, and outlines necessary and critical steps needed to further astrovirus research.

## 2. Astrovirus Disease

Within the years following the discovery of HAstV, eight distinct genotypes (HAstV-1–8, also referred to as classical or canonical human clades) were identified, with infections caused by HAstV-1 shown to be the most prevalent worldwide [34,35,36,37,38]. In the late 2000s, through pathogen discovery investigations of diarrheal outbreaks, two divergent HAstV genotypes were discovered: HAstV-MLB (Melbourne) clade containing at least three strains (MLB1, MLB2, MLB3) and the HAstV-VA/HMO (Virginia/Human-Mink-Ovine-like) clade containing at least five strains (VA1, VA2, VA3, VA4, VA5). The HAstV-MLB and HAstV-VA/HMO viruses have been designated as non-canonical human genotypes. HAstV-MLB1 was initially discovered in pediatric stool samples from Australia [39,40,41], whereas HAstV-MLB2 and HAstV-MLB3 were discovered in India [42,43]. The HAstV-VA1–VA3 viruses were first discovered in an outbreak of gastroenteritis in Virginia, USA [44,45]; HAstV-VA4 in a cohort of Indian children with diarrhea [43]; and HAstV-VA5 in a cohort in Gambia [46]. The prevalence of the non-canonical viruses varies greatly according to geographic location [6], and their association with clinical disease remains somewhat of a mystery in comparison to the canonical strains.

Signs of HAstV last 2 to 4 days, and consist of watery diarrhea that can be less commonly accompanied by fever, headaches, abdominal pain, and anorexia [1,47,48]. However, many of the infections in healthy children and adults tend to be asymptomatic [37]. The consequence of these asymptomatic cases on the epidemiology and transmission of the virus remain unclear. Further research is needed to understand the clinical disease associated with the different human astrovirus genotypes.

Astrovirus infections are of clinical concern in the immunocompromised population due to their increased severity of symptoms and extra-gastrointestinal involvement [6]. HAstV has also been suggested to be the causative agent in encephalitis and meningitis, which was brought to light in a case report of a 15-year old boy with X-linked agammaglobulinemia by Quan et al. [33]. The patient was admitted to a psychiatric ward due to cognitive decline, became comatose, and died 71 days post admission. RNA was extracted from the patient’s biopsy and postmortem samples, and sequenced and amplified with virus-specific primers, but results were negative. As an alternative approach, next-generation sequencing (NGS) was utilized to identify the presence of HAstV in the biopsy samples. Consequently, there have now been nine cases of astrovirus-associated encephalitis and meningitis reported in predominately immunocompromised patients, although one case was in an individual without overt immunosuppression [33,49,50,51,52,53,54,55]. Interestingly, in eight out of the nine cases, a non-canonical strain was detected in patient samples. NGS identified HAstV-VA1 in a nasopharyngeal specimen from a child with an acute respiratory infection in Tanzania, but it is unclear if HAstV-VA1 was responsible for the respiratory disease [56]. These studies suggest that astroviruses have systemic potential, reiterating the importance of studying astrovirus pathogenesis of both canonical and non-canonical subtypes, especially since infections caused by non-canonical viruses do not appear to be rare in immunocompromised populations. In a cohort of immunocompromised pediatric oncology patients, while HAstV-1 was identified in 50% of samples, HAstV-VA2 and HAstV-MLB1 were also present in 21% and 13% of samples, respectively [57]. However, it was noted that the PCR screening used in this study was unable to detect other members of the HAstV-VA and HAstV-MLB clades, meaning that these genotypes may be even more prevalent than reported. With improved molecular techniques for detection and diagnosis, surveillance efforts will afford a better understanding of prevalence.

There are many parallels that can be drawn between human and animal astrovirus disease, but the area remains understudied. Like humans, many of the animals infected with astrovirus exhibit diarrhea, including turkeys [22], chickens [23], calves [24], lambs [25], piglets [21], and deer [20]. However, there is a subset of animals that are asymptomatic [12,19,21,24]. Much like the human astroviruses, the impact of the asymptomatic infection on epidemiology and transmission in animal astrovirus remains unclear. Chicken astrovirus has been associated with “white chick” condition that causes a decrease in hatch-rate and an increase in chick mortality and weakness [58,59]. Along with this, there is evidence of astrovirus-associated encephalitis and meningitis in mink and cows [31,32] and respiratory disease in calves [60]. As with the human viruses, improved molecular techniques would lead to a better understanding of prevalence and genotype-specific disease.

## 3. Cell Culture and Animal Models

One of the major barriers to understanding astrovirus pathogenesis is the lack of suitable cell culture systems for the majority of the genotypes. The classical HAstV strains replicate in a variety of cells lines, with human colon carcinoma cells type 2 (Caco-2) being the most widely used [61]. No cell culture systems have been identified for the non-canonical HAstV-MLB and HAstV-VA genotypes, or the majority of animal and avian viruses. Unfortunately, until astrovirus cellular receptor(s) are identified, we remain unable to create suitable cell culture systems.

Numerous groups are working to identify cellular binding proteins. Crystallization of the human HAstV-8 and turkey astrovirus type 2 (TAstV-2) capsid and spike proteins [62,63,64,65,66] showed conserved polysaccharide binding motifs [65]. The addition of heparin, heparin sulfate, or dextran sulfate partially blocked HAstV-8 infectivity of Caco-2 cells [66]. However, pretreatment of Caco-2 cells with chondroitinase and heparinase did not significantly reduce HAstV-1 infection in the same cells [63], suggesting that there may be strain- and/or genotype-specific differences. Identifying the astrovirus receptor will be a key discovery, and will possibly allow the creation of transgenic animal models to further study pathogenesis and astrovirus-associated disease.

In addition to the lack of cell culture systems, there is no animal model for HAstVs. To date, turkey poults remain the only well-defined “small” animal model available to study astrovirus pathogenesis in vivo. Research done using the turkey poult model has shown that—unlike other gastrointestinal viruses—astrovirus did not cause diarrhea by damaging the intestinal epithelium and/or eliciting an inflammatory response [67]. Rather, infection may lead to diarrhea by inducing sodium malabsorption [68], and possibly by disrupting the intestinal epithelial barrier [69].

**Figure 2:**
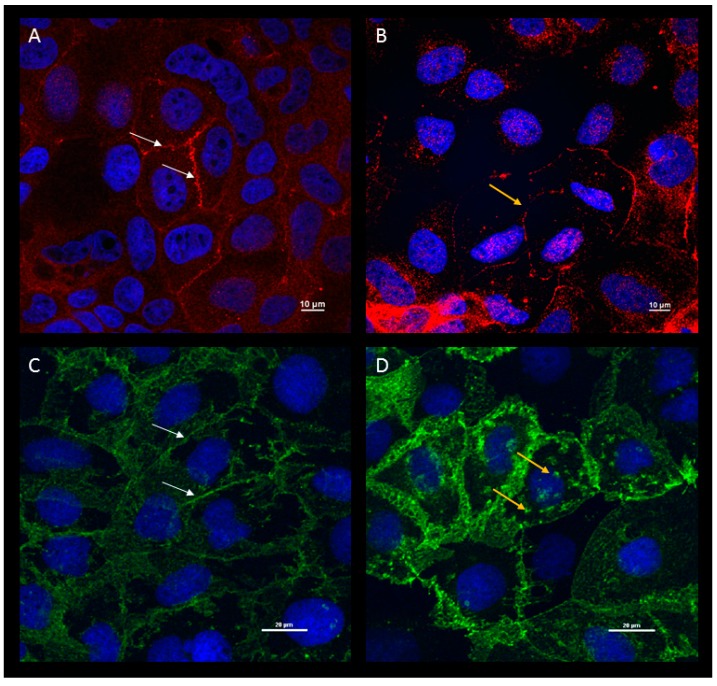
HAstV-1 infection causes a loss of occludin and relocalization of e-cadherin. Mock (**A**,**C**) or HAstV-1-infected (**B**,**D**) human colon carcinoma cells type 2 (Caco-2) were stained for occludin (red, **A**,**B**), e-cadherin (green, **C**,**D**) or DAPI (blue) 24 h post-infection (hpi) and imaged by confocal microscopy. White arrows show normal junction appearance, while yellow arrows indicate apparent relocalization junction proteins. Magnification bars shown inset.

HAstV-1 infection of Caco-2 cells results in increased barrier permeability by disrupting tight junctions [30]. Tight junctions are highly regulated cell–cell associations that help maintain cell polarity and prevent the free passage of macromolecules and microorganisms from one side of the epithelium to the other [70]. Junctions are multiprotein domains composed of transmembrane proteins, such as occludin and claudins, which interact with cytosolic adapter proteins, like zonula occludens (ZO-1). These interactions connect the cell membrane with the actin cytoskeleton. Disruptions of the tight junction can result in altered ion and/or solute exchange, increasing fluid to the lumen of the intestine and inducing diarrhea. Astrovirus has been shown to increase epithelial cell permeability upon infection of Caco-2 cells in vitro, possibly due to a reduction in actin fibers [30], reduction of occludin from the junctional complex [30], and redistribution of e-cadherin 24 h post-infection (hpi; Figure 2).

Intriguingly, this disruption of tight junctions did not require infectious virus. Recombinant HAstV-1 capsid protein also increased barrier permeability in vitro [30], and oral administration of recombinant TAstV-2 capsid protein alone induced diarrhea, caused relocalization of transporters, and disrupted intestinal permeability in turkey poults [69], suggesting that the astrovirus capsid protein may act as a type of enterotoxin. We hypothesize that binding alone (to a yet unknown receptor) or entry may trigger a signal transduction cascade, resulting in dysregulation of intestinal tight junctions. However, what this disruption means for the viral life cycle or disease pathogenesis requires more research. Studies are also underway to identify the cellular mechanisms leading to disruption as well as the region(s) of the capsid involved in activity, including the identification of cellular receptor(s).

The use of the Caco-2 in vitro model also led to the discovery that HAstV infection does not induce a strong interferon (IFN) response [71], yet exogenous IFN-β effectively reduced HAstV infection [71,72], and pretreatment significantly reduced HAstV-1-increased barrier permeability [72]. Similar results of decreased infection and less barrier permeability were seen in vivo using interferon-alpha/beta receptor (IFNAR) knockout mice [72]. This indicates that type I interferons play a crucial role in protection against astrovirus replication, and potentially protect the intestinal integrity during infection.

While turkey poults are the best-characterized small-animal model for astrovirus-induced gastroenteritis, they are not ideal. TAstV is a member of *Avastrovirus*, making it genetically distinct from human astroviruses. Thus, disease pathogenesis of the two may not be comparable. Additionally, there are few commercially available reagents (including antibodies) for most cellular proteins [69]. Given the lack of reagents, the genetic differences in avian and mammalian astroviruses, and the challenge of working with the turkey model, a mammalian model would be better suited to study astrovirus pathogenesis.

In 2012, two novel murine astroviruses (MuAstV; STL1 and STL2) were identified in mice housed in a specific-pathogen-free facility at Washington University in Saint Louis, MO, USA [73]. Subsequent analysis revealed the presence of at least two additional viruses (STL3 and STL4) in this colony. Yokoyama et al. showed that MuAstV was shed at high levels in the feces of immunocompromised mice, and MuAstV genomic RNA could be detected in numerous organs [73]. The virus was highly infectious, as cohousing resulted in transmission to naïve animals. Similarly, we isolated a MuAstV strain most similar to STL4, developed purification methods, and demonstrated that oral inoculation of 3 to 6 weeks of age C57Bl/6 mice resulted in high viral titers in the feces beginning at 2 days post-infection (dpi) that continued through 53 dpi [74]. This is similar to some strains of murine norovirus, where mice exhibit low-level persistent infection with virus shed in the feces for extended periods of time and the potential to go systemic in the absence of overt disease [75,76]. Unlike TAstV-2-infected turkey poults, mice did not exhibit diarrhea as a symptom of MuAstV infection. This is unsurprising, given that colitis must be induced for diarrhea to occur in mice [74,77], and preliminary studies suggest that MuAstV infection does not cause intestinal damage. However, MuAstV did increase intestinal permeability, suggesting that increased permeability may be independent of diarrhea in the mouse model. More studies are needed to understand the correlation (if there is any) between increased permeability and diarrhea, as well as the ability of the capsid protein alone to induce clinical disease.

Finally, something to consider: our group and others showed that MuAstV is not only present in many strains of commonly used laboratory mice, but is persistently shed at high levels, and may be systemic [73,78]. What does this mean for biomedical research? Does an underlying, asymptomatic MuAstV infection impact other infectious disease, immunity, or cancer studies? What about the microbiome? Our preliminary data suggests that MuAstV infection does change the microbiome, but what happens if we remove the virus? While we do not yet know the impact of endemic MuAstV infection, studies from MNV suggest that it could have broad implications on biomedical research. For example, we know that underlying MNV infection can alter the disease progression in bacterially-induced inflammatory bowel disease [79], can induce intestinal inflammation similar to Crohn’s disease in susceptible mice strains [80], and most intriguingly, may be able to replace the beneficial functions of the gut microbiome [81,82]. All of these and many other questions require answers, and therefore it is an exciting time to study astroviruses.

## 4. Conclusions

Although there is still much work to be done regarding astrovirus pathogenesis, significant progress has been made in recent years. The identification of HAstV-MLB and HAstV-VA genotypes show that astrovirus infections are more common than was once thought. Along with the discovery of new astroviruses, a greater appreciation for the virus’ potential to go systemic and cause encephalitis and meningitis in immunocompromised and immunocompetent hosts has been developed. However, it has also become clear that astrovirus causes diarrhea in an unconventional way. Rather than causing inflammation and cell death in the intestinal tract, astrovirus disrupts tight junctions, allowing an influx of water, resulting in diarrhea. Along with this, we now know from a turkey poult model that the viral capsid alone is capable of invoking diarrhea in vivo. With all of this new knowledge, we still face challenges, the most prominent being the lack of a well-characterized small-animal model. As this model becomes more developed and standardized, astrovirus pathogenesis can be further elucidated and eventually used for vaccination, therapeutic studies, and furthering our knowledge on possible zoonotic transmission. A major question is whether an animal model for HAstV strains is possible. Our recent study demonstrating that non-human primates harbor human astroviruses suggest that it may be a possibility in the future [28].
